# Prevalence of the American College of Rheumatology classification criteria in a group of 162 systemic lupus erythematosus patients from Croatia

**DOI:** 10.3325/cmj.2012.53.149

**Published:** 2012-04

**Authors:** Mislav Cerovec, Branimir Anić, Ivan Padjen, Nada Čikeš

**Affiliations:** Division of Clinical Immunology and Rheumatology, Department of Internal Medicine, University of Zagreb School of Medicine, University Hospital Center Zagreb, Zagreb, Croatia

## Abstract

**Aim:**

To identify systemic lupus erythematosus (SLE) patients diagnosed and treated at the outpatient clinic of our Division fulfilling at least four American College of Rheumatology (ACR) classification criteria at the time of the study, to determine the prevalence of each of the criteria at three different time points, and to compare the data with similar studies.

**Methods:**

We performed retrospective and descriptive analysis of medical records of 162 patients fulfilling at least 4 ACR criteria. Classification criteria were counted and the frequency of each criterion was identified at three different time points: disease onset, time of diagnosis, and the time when the study was conducted.

**Results:**

At diagnosis and at the time when the study was conducted there were 3.8 and 5.4 fulfilled classification criteria, respectively. The most common criterion at the time of the disease onset was arthritis (52.6%); at the time of diagnosis it was positive antinuclear antibody (ANA) titer (88.0%); and at the time when the study was conducted it was positive ANA titer (95.7%), immunologic disorder (89.5%), arthritis (71.0%), hematologic disorder (70.4%), malar rash (61.7%), and photosensitivity (51.9%).

**Conclusion:**

The prevalence of ACR criteria in our patients is similar to that in other studies, especially those involving Caucasian patients. Our results confirm the value of the ACR criteria in patients with an already established diagnosis. This is the first study on the prevalence of disease manifestations among Croatian patients with SLE.

Systemic lupus erythematosus (SLE) is a chronic inflammatory disease affecting a number of organs and organ systems (1-3). The wide spectrum of clinical features among patients with SLE, progress of medical research and, consequently, better understanding of the disease led to a need for a unique classification of disease manifestations for research purposes. The American College of Rheumatology (ACR) developed classification criteria for SLE in 1982 based on a study conducted on 177 patients with SLE and 162 healthy individuals (4). The criteria have been accepted throughout the world, with only one revision in 1997 (3). A patient is considered to have SLE if at least four criteria are cumulatively fulfilled, with a high sensitivity and specificity (both 96%) in patients with an already established diagnosis (4). In addition to their primary role in clinical research, the criteria may also be used as a helpful tool for establishing the diagnosis, since they comprise the majority of specific disease manifestations.

Apart from a number of case reports and case series, no systematically presented data are available on the prevalence of clinical and laboratory manifestations of the disease among Croatian patients (5-8). The aim of this study is 1) to identify SLE patients diagnosed and treated in the outpatient clinic of our Division fulfilling at least four ACR classification criteria at the time of the study, 2) to determine the prevalence of each of the fulfilled criteria at three different time points: at disease onset, the time of diagnosis, and the time when the study was conducted, and 3) to compare the data with similar studies.

## Patients and methods

This is a retrospective and descriptive study of the prevalence of the ACR criteria for classification of SLE among patients of the Division of Clinical Immunology and Rheumatology, University Hospital Centre Zagreb, diagnosed or suspected with SLE. We used the 1982 revised ACR classification criteria and their 1997 updated version (3,4). In the context of this study and everyday clinical practice, the term clinical diagnosis does not necessarily imply that 4 or more ACR criteria have been fulfilled (1-3).

Data for this study were retrieved from patient records of the Division’s outpatient clinic and analyzed between September and December 2004. At the time when the study was conducted, the Division kept approximately 25 000 medical records of patients suffering from any form of inflammatory rheumatic disease, with at least one visit to the outpatient clinic in the 25-year history of the Division. Patients were diagnosed, treated, and followed-up by certified rheumatologists. The Division offers general rheumatology service for the city of Zagreb and its surroundings, as well as northwestern Croatia. It also serves as a center of excellence in connective tissue diseases, offering its service to patients from the whole country, and a large number of patients from Bosnia and Herzegovina.

The first and second review of the medical records were performed by a board of the Division’s specialists working in pairs (a total of 6 rheumatologists). The reviewers identified medical records of 1415 patients with a clinical or suspected diagnosis of SLE. These medical records were subsequently evaluated.

The next inclusion criterion was patients’ visit to the outpatient clinic within three years before the beginning of the study, as an indicator of a continuous follow-up. Patients not living in Croatia and/or non-Croatian citizens were excluded from the study. We formed a group of 333 patients with a clinical diagnosis or suspicion of SLE, who were Croatian citizens and residents under regular follow-up by rheumatologists of the Division. Their medical records were subsequently reanalyzed: the cumulative frequency of each of the ACR criteria at the time of our study was determined. Patients fulfilling fewer than four criteria were excluded from further analysis. We finally identified 162 patients fulfilling at least four ACR criteria at the time when the study was conducted. Having identified the 162 patients, we counted the criteria that each patient fulfilled at three different time points: (a) at the disease onset, (b) at the time of diagnosis, and (c) at the time when the study was conducted. We determined the prevalence of each of the classification criteria at these time points. Despite the systematic approach, loss of data was observed due to the retrospective nature of the study and use of patients’ medical records for data retrieval. The loss of data was especially observed for the time of disease onset and the time of diagnosis.

The results were put into the context of similar data available in the relevant literature. Calculations (including percentages and medians) were performed using Microsoft Excel 2003 (Microsoft Corporation, Redmond, WA, USA).

## Results

At the time when the study was conducted, 162 patients fulfilled at least four ACR criteria (Table 1). There were 145 female and 17 male patients, between 19 and 81 years of age. The median age in the studied group was 47. Age range for men was between 22 and 74 years (median, 47) and age range for women was between 19 and 81 years (median, 47). At the time when the study was conducted there were 5.4 fulfilled classification criteria, compared to only 3.8 at the time of diagnosis.

**Table 1:** The prevalence of the American College of Rheumatology (ACR) classification criteria for systemic lupus erythematosus (SLE) in 162 patients fulfilling at least 4 criteria at the time when the study was conducted (data available for 162 patients), at the time of establishing the diagnosis, and at disease onset (data available for 125/162 and 78/162 patients, respectively)

		No (%) of patients fulfilling each criterion at:		
	ACR classification criteria for SLE	SLE onset	SLE diagnosis	time when the study was conducted
1	Malar rash	21 (26.9)	48 (38.4)	100 (61.7)
2	Discoid rash	4 (5.1)	7 (5.6)	24 (14.8)
3	Photosensitivity	18 (23.1)	43 (34.4)	84 (51.9)
4	Oral ulcers	11 (14.1)	18 (14.4)	27 (16.7)
5	Arthritis	41 (52.6)	69 (55.2)	115 (71.0)
6	Serositis	10 (12.8)	19 (15.2)	28 (17.3)
a	Pleuritis	6 (7.7)	13 (10.4)	17 (10.5)
b	Pericarditis	2 (2.6)	9 (7.2)	11 (6.8)
c	Sub-criterion not determined	2 (2.6)	3 (2.4)	5 (3.1)
7	Renal disorder	6 (7.7)	19 (15.2)	42 (25.9)
8	Neurologic disorder	2 (2.6)	3 (2.4)	9 (5.6)
a	Seizures	2 (2.6)	3 (2.4)	6 (3.7)
b	Psychosis	0 (0.0)	0 (0.0)	3 (1.9)
9	Hematologic disorder	29 (37.2)	67 (53.6)	114 (70.4)
a	Hemolytic anemia	3 (3.8)	11 (8.8)	14 (8.6)
b	Leukopenia	18 (23.1)	43 (34.4)	75 (46.3)
c	Lymphopenia	5 (6.4)	26 (20.8)	53 (32.7)
d	Thrombocytopenia	13 (16.7)	19 (15.2)	26 (16.0)
e	Sub-criterion not determined	0 (0.0)	1 (0.8)	5 (3.1)
10	Immunologic disorder	12 (15.4)	74 (59.2)	145 (89.5)
a	Anti-DNA	10 (12.8)	68 (54.4)	136 (84.0)
b	Anti-Sm	NA*	NA*	NA*
c	Antiphospholipid antibodies	3 (3.8)	32 (25.6)	45 (27.8)
d	Sub-criterion not determined	1 (1.3)	3 (2.4)	8 (4.9)
11	Antinuclear antibody	18 (23.1)	110 (88.0)	155 (95.7)
				

*NA – data not available.

Data on the frequency of subcriteria were insufficient for the following ACR criteria: serositis, hematologic and immunologic disorder (Table 1). In addition, due to the retrospective nature of the study, complete data for all patients in the observed group were not available at the time of disease onset and diagnosis. Data for the time of disease onset were available for only 78 out of 162 patients and data for the time of diagnosis were available for 125 patients (Table 1).

The most frequently observed classification criteria at disease onset were mucocutaneous manifestations (malar and discoid rash, photosensitivity, oral ulcers) and arthritis. At the time of diagnosis, the most prevalent classification criterion was a positive ANA titer, although less frequently observed than at the time when the study was conducted. Criteria with the highest observed prevalence at the time when the study was conducted were from the group of impaired laboratory findings, ie, hematologic (cytopenias) and immunologic (antinuclear antibodies [ANA], anti-DNA antibodies and/or anti-Sm antibodies and/or lupus anticoagulant, and/or anticardiolipin antibodies) disorders. The most frequently fulfilled clinical criteria were arthritis and malar rash.

## Discussion

Our data on the prevalence of the ACR criteria at the time when the study was conducted are similar to the findings of other authors. In addition, the total number of patients involved in this study is comparable with some of the major studies on the prevalence of ACR criteria and other clinical manifestations among patients diagnosed with SLE (Table 2) (4,9-11).

**Table 2:** Comparison of the prevalence of each of the American College of Rheumatology (ACR) classification criteria between our study (Croatia) and similar studies conducted in patients diagnosed with systemic lupus erythematosus (SLE)

The percentage of patients with the criterion	USA (4)	Europe (9)	Germany (10)	Norway (11)	Croatia
Malar rash	57.0	26.4	67.0	51.0	61.7
Discoid rash	18.0	5.4	44.0	16.0	14.8
Photosensitivity	43.0	18.7	72.0	49.0	51.9
Oral ulcers	27.0	8.9	33.0	2.0	16.7
Arthritis	86.0	41.3	71.0	87.0	71.0
Serositis	56.0	12.9	30.0	42.0	17.3
Renal disorder	51.0	22.2	42.0	22.0	25.9
Neurologic disorder	20.0	13.6	21.0	11.0	5.6
Hematologic disorder	59.0	12.8	97.0	46.0	70.4
Immunologic disorder	85.0	NA*	96.0	67.0	89.5
Antinuclear antibody	99.0	90.5	96.0	100.0	95.7
Number of patients	177	1000	338	55	162

*NA – data not available.

The ACR criteria for classification of SLE were developed on the basis of the most frequent and specific clinical and laboratory disease manifestations observed in 177 patients diagnosed with SLE (4). A patient is considered to have SLE if at least 4 of the 11 criteria are fulfilled, serially or simultaneously, during any interval of observation (3,4). For patients with an already established diagnosis, the sensitivity and specificity of the ACR criteria are both very high – 96% (3). Although the criteria are widely used in the research setting, it is still not clear whether establishing the diagnosis of SLE should solely rely on the strict fulfillment of at least four criteria at such an early stage of the disease course.

Our results confirm the value of the criteria in patients with an already established diagnosis. On the other hand, they reveal their questionable value in the early stages of the disease course, including the time of diagnosis – the sensitivity of the criteria may be much lower than in the patients with an already established diagnosis. There was an obvious difference between the prevalence of ACR criteria at the disease onset and at more developed stages of the disease course. This can be attributed to the retrospective nature of our study and the reliance of the available data on the criteria at the disease onset primarily on patients’ histories. It is noteworthy that the presence of only two classification criteria can be identified on the basis of a patient’s history: photosensitivity and pleuritis (3,4).

Analysis of the prevalence of fulfilled classification criteria at the time of diagnosis implicates that the diagnosis established in the clinical setting does not completely depend on the fulfillment of the ACR criteria. The number of fulfilled ACR criteria at the time of diagnosis was 3.8, revealing that a considerable percentage of patients were clinically diagnosed with SLE yet not fulfilling four ACR criteria. A variety of different clinical and laboratory findings should be taken into account when establishing the diagnosis of SLE in everyday practice. There are symptoms and signs occurring at the beginning of the disease course that do not fully meet the ACR criteria (ie, arthralgia without arthritis, oral ulcers not observed by a physician but only as a part of a patient’s history) (12,13). Furthermore, there is a myriad of clinical and laboratory parameters that are not included in the classification criteria but can, nevertheless, indicate the presence of disease or disease activity (ie, fever, various forms of skin eruptions or mucosal changes, different neurological manifestations, complement deficiency, human leukocyte antigens associated with SLE) (1,2).

Cumulative fulfillment of 4 or more ACR classification criteria assures that patients in an observed group are really suffering from SLE. However, it should be noted that the presence of multiple classification criteria does not necessarily mean a severe course of the disease. It is not possible to assess SLE disease activity using the classification criteria – different disease activity indexes have been developed for this purpose (14-18).

We faced several limitations and biases in our study. The retrospective design led to a loss of a considerable number of patient data on the prevalence of ACR criteria at the time of diagnosis and even more at the disease onset, as well as on the prevalence of several subcriteria. Additionally, our analysis was a single-center one. Although the largest number of Croatian SLE patients is followed-up by the Division, other institutions’ patients should have also been included in the study in order to obtain results more representative for the whole country.

In conclusion, the prevalence of ACR criteria at the time when the study was conducted is similar to other studies, especially those involving Caucasian patients. This confirms the value of the ACR criteria in patients with an already established diagnosis. However, it seems that the ACR criteria alone cannot be used as diagnostic criteria due to a variety of clinical and laboratory findings that should be considered when establishing the diagnosis but are not included in the ACR criteria. This study can serve as a promising step toward a more thorough research of SLE disease manifestations, especially prospective follow-up.
